# Ultrasound-guided posterior quadratus lumborum block can reduce postoperative opioid consumption and promote rapid recovery in patients undergoing sutureless laparoscopic partial nephrectomy: A triple-blind, randomized, controlled study

**DOI:** 10.3389/fonc.2022.969452

**Published:** 2022-10-06

**Authors:** Youzhuang Zhu, Zhichao Li, Shangyuan Qin, Hao Xu, Jianshuai He, Fang Sheng, Qin Zhao, Yihan Kang, Xin Gao, Si Li, Jun Chai, Lina Chen, Weiwei Wang

**Affiliations:** ^1^ Department of Anesthesiology, The Affiliated Hospital of Qingdao University, Qingdao, China; ^2^ Department of Anesthesiology, Shengjing Hospital of China Medical University, Shenyang, China; ^3^ Department of Anesthesiology, Shandong provincial Qianfoshan Hospital, Jinan, China; ^4^ Department of Anesthesiology, Weihai Municipal Hospital, Cheeloo College of Medicine, Shandong University, Weihai, China

**Keywords:** quadratus lumborum block, laparoscopic partial nephrectomy, postoperative analgesia, quality of recovery, ultrasonography

## Abstract

**Purpose:**

We hypothesized that posterior quadratus lumborum block would reduce postoperative opioid consumption and improve the quality of recovery in patients undergoing sutureless laparoscopic partial nephrectomy.

**Materials and methods:**

The study included 60 patients, ages 18−65 with American Society of Anesthesiologists scores of I-II scheduled for elective sutureless laparoscopic partial nephrectomy. Before general anesthesia, 60 participating patients were randomly allocated to receive a 30-ml injection posterior to the quadratus lumborum muscle with either 0.375% ropivacaine (*n* = 30) or normal saline (*n* = 30). The primary outcomes included cumulative opioid consumption within 12 h postoperatively and quality of postoperative recovery at 48 h. Secondary outcomes included the Numerical Rating Scale (NRS), opioid consumption by period, first time to press the analgesic pump, number of patients needing rescue analgesia, blood glucose and cortisol concentrations, early postoperative recovery indicators, and adverse events.

**Results:**

There were 48 patients included in the final analysis. The intervention group had lower cumulative consumption of sufentanil within 12 h postoperatively and higher quality of postoperative recovery scores at 48 h postoperatively compared with the control group (*p* < 0.001). The NRS at resting and movement of the intervention group was lower at 0 h, 6 h, and 12 h after surgery than in the control group (*p* < 0.05). At prespecified intervals (0 to 2 h, 2 to 6 h, 6 to 12 h, 12 to 24 h, and 24 to 48 h) after surgery, the intervention group had lower consumption of sufentanil compared with the control group (*p* < 0.05). The intervention group took longer to press the analgesic pump for the first time within 48 h after surgery compared with the control group (*p* < 0.001). The postoperative blood glucose and cortisol concentrations in the intervention group were lower than in the control group (*p* < 0.05). The times to first excretion, ambulation, and discharge were shorter in the intervention group compared with the control group (*p* < 0.05). There was no significant difference in adverse events between the two groups.

**Conclusions:**

Our trial demonstrated that patients who received posterior quadratus lumborum block had significantly lower opioid consumption within 12 h postoperatively and had a better quality of recovery at 48 h postoperatively. Therefore, we recommend posterior quadratus lumborum block as an option for postoperative analgesia in patients undergoing sutureless laparoscopic partial nephrectomy.

**Trial Registration:**

http://www.chictr.org.cn, identifier ChiCTR2100053439.

## Introduction

Renal cell carcinoma is a common malignant tumor of the urinary system, and the incidence of renal cell carcinoma has risen in recent years (1). Laparoscopic partial nephrectomy is the primary surgical method for treating renal cell carcinoma. It has the advantages of less trauma and faster recovery. However, intraoperative procedures include abdominal wall incision, tissue damage, inflation effect of pneumoperitoneum, traction and suture of kidney and ureter, and persistent stimulation of the peritoneum by residual carbon dioxide after surgery cause varying degrees of postoperative somatic and visceral pain. It is worth mentioning that the laparoscopic partial nephrectomy selected in this study is a new surgical procedure−sutureless laparoscopic partial nephrectomy (sLPN). The sLPN is different from the previous traditional surgery, without the need to suture the renal basal and parenchymal layers, and uses monopolar coagulation combined with hemostatic glue to stop bleeding. The sLNP dramatically reduces the damage to renal parenchyma caused by direct suture and warm ischemia and ischemia-reperfusion injury caused by prolonged suture time. This procedure may be beneficial for protecting renal function (2). However, according to our retrospective case observation, the sLPN is accompanied by higher postoperative opioid consumption and pain than traditional surgery. This situation may be attributable to the coagulation injury caused by prolonged and large-area monopolar coagulation hemostasis during surgery, which aggravates postoperative visceral pain. Therefore, patients undergoing sLPN had higher requirements for postoperative analgesia than those who underwent traditional surgery.

Patient-controlled intravenous analgesia (PCIA) with opioids is commonly used postoperatively for laparoscopic partial nephrectomy. However, this single analgesic mode has a poor analgesic effect and low patient compliance. There are many adverse reactions caused by opioids, such as respiratory depression, nausea and vomiting, urinary retention, and delayed recovery of gastrointestinal function (3). The popularization of enhanced recovery after surgery (ERAS) makes postoperative multimodal analgesia superior. It emphasizes the combined application of different drugs or methods acting on the pain conduction pathways to achieve optimal analgesic effects and minimize the dosage of opioids, thereby reducing the occurrence of adverse reactions. In recent years, the ultrasound-guided regional block has been an essential part of multimodal analgesia. Several studies (4–6) have shown that regional block can reduce postoperative opioid consumption and pain scores, reduce the postoperative bed rest time and hospitalization time, and improve patient clinical outcomes. Regional block has become a hot topic of postoperative analgesia treatment. However, there is still a lack of corresponding clinical research on the analgesic mechanism of the regional block, its impact on the stress response, and quantitative assessment of its impact on early postoperative recovery (7).

The quadratus lumborum block (QLB) is a trunk nerve block technique. Studies have reported that local anesthetics at the injection site after QLB can diffuse cranially through the thoracolumbar fascia to the thoracic paravertebral space, infiltrating the sympathetic thoracic trunk and somatic nerves (8, 9). Therefore, it can block not only somatic pain but also block visceral pain. In different puncture paths, QLB can be divided into lateral QLB, posterior QLB, anterior QLB, and intramuscular QLB. Some studies (10, 11) showed that lateral QLB and anterior QLB successfully reduced pain scores and opioid consumption after laparoscopic nephrectomy and recommended it as an option for analgesia after this procedure. The role of posterior QLB in laparoscopic nephrectomy is contradictory (12, 13), and the specific injection targets (posterolateral, posteromedial, lumbar interfascial triangle) have not been described in detail. To our knowledge, this study is the first time a posterior QLB technique has been applied to patients undergoing sLPN. Anatomic study and staining experiments have confirmed that local anesthetics for posterior QLB have been observed to spread predictably in the thoracic paravertebral space. The lumbar interfascial triangle was identified as the optimal injection site for posterior QLB (14). Therefore, in this study, posterior QLB was performed at the lumbar interfascial triangle. We hypothesized that posterior QLB could reduce postoperative opioid consumption and pain scores and promote the quality of postoperative recovery. At the same time, we hope this study can provide a reference and basis for selecting reasonable postoperative analgesic regimens for patients with sLPN.

## Methods

### Trial design and setting

This was a single-center, block-randomized, triple-blind controlled study approved by the Ethics Committee of Shengjing Hospital of China Medical University (2021PS026K) and registered at the Chinese Clinical Trial Registry (ChiCTR2100053439). This study was conducted in full compliance with the Consolidated Standards of Reporting Trials (CONSORT) statement and the Declaration of Helsinki. The Ethics Committee and Clinical Experiment Management Department of Shengjing Hospital of China Medical University monitor clinical trials according to the rules formulated by the hospital.

### Participants

This study was conducted at Shengjing Hospital of China Medical University from November 2021 to April 2022, and 60 subjects were enrolled. The inclusion criteria were that patients were diagnosed with renal tumors and underwent elective sLPN, aged 18−65 years, male or female, body mass index (BMI) < 28 kg/m^2^, and American Society of Anesthesiologists (ASA) physical status classification I or II (I [healthy], II [mild systemic disease]). Exclusion criteria included severe cardiovascular and cerebrovascular diseases, such as Grade-3 hypertension, unstable angina, severe arrhythmia, severe heart valve disease, cerebral infarction and cerebral hemorrhage, neurological disease or somatosensory abnormalities, history of chronic pain and long-term use of pain medication, mental illness, unable to understand and cooperate, skin infection near the puncture site, local anesthetic allergy, abnormal renal function (glomerular filtration rate < 90 ml/min), intraoperative blood transfusion, intraoperative use of exogenous hormone drugs such as dexamethasone and methylprednisolone, diabetic (fasting blood glucose ≥ 126 mg/dl, or 2-hour blood glucose after oral 75 g glucose tolerance test (OGTT) ≥ 200 mg/dl, or hemoglobin (A1C) ≥ 6.5%), adrenal hyperfunction (Cushing’s syndrome, aldosteronism) or hypofunction (primary or secondary), and operation time > 3 h.

### Randomization and blinding

Subjects were numbered 1−60 in the order of enrollment. It was planned to divide subjects into two groups in a 1:1 ratio, with the block length set to 4. A string of random numbers was generated by the Statistical Package for Social Science, the numbers 1-6 were recorded in sequence from the random number list, and then the blocks were arranged according to the random registered numbers. Randomization and block assignment was performed by the Clinical Trials Department, which was not involved in other parts of the trial. The randomization scheme was placed in sealed opaque envelopes. Subjects were randomly assigned to receive unilateral posterior QLB with either 30 ml 0.375% ropivacaine (intervention group) or 30 ml isotonic saline (control group). The choice of dose and concentration of ropivacaine was based on the pharmacokinetics and pharmacodynamics of ropivacaine, and the Department of Pharmaceutical Management recommended the single injection dose of ropivacaine. Two anesthesiologists (YHK and SYQ) opened the randomization envelope, then prepared a 30-ml syringe in a separate room according to the random allocation scheme of the sealed envelope, and handed it to the anesthesiologist (JC) who performed the nerve block. The syringe was filled with 30 ml 0.375% ropivacaine or 30 ml 0.9% isotonic saline. The blinding process was strictly ensured, and interviews related to the trial drug were not allowed. All syringes had the same appearance. To ensure blinding to the anesthesiologist and subjects, each subject had an ultrasound-guided posterior QLB using the drug from a prepared syringe. Throughout the study, the anesthesiologists who performed nerve blocks, nursing staff, and surgeons were blinded to the assignment and treatment of subjects.

### Posterior quadratus lumborum block procedure

Patients were placed in the lateral position in the nerve block room with the operative side up. The posterior QLB was performed under ultrasound guidance (TE7 Ultrasound System, Mindray Medical, Shenzhen, China). The puncture site was fully exposed and disinfected with an iodophor disinfectant. The convex array low-frequency (1.3–6 MHz) ultrasound probe was covered with a sterile plastic protective sleeve (Sterile Ultrasound Probe Cover, LOOKMED Medical Devices, Changzhou, China). The ultrasound probe was placed laterally on the midaxillary line between the costal arch and the iliac spine. The three layers of muscles in the abdomen, namely the external oblique muscle, internal oblique muscle, and transverse abdominis muscle, were visualized on the ultrasonic display screen. The ultrasound probe was gradually moved backward until the quadratus lumborum, erector spinae, latissimus dorsi muscles, and thoracolumbar fascia (TLF) were visualized under ultrasound. The TLF consisted of anterior, middle, and posterior layers. The anterior layer of the TLF covers the front of the quadratus lumborum, the middle layer of the TLF separates the quadratus lumborum and the erector spinae, and the posterior layer of the TLF covers the surface of the erector spinae. In the lumbar segment, the posterior layer of the TLF extends from the medial spinous process to the lateral border of the erector spinae, where it fuses with the medial layer of the TLF to form the lateral raphe, which is a column of dense fascial tissue, extending from the iliac crest to the 12th rib. The posterior layer of the TLF consists of a superficial layer and a deep layer, of which the deep layer is called the paraspinal retinacular sheath (PRS).

**Figure 1:**
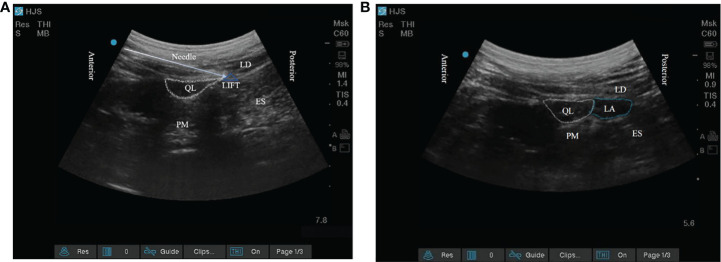
**(A)** The target of posterior quadratus lumborum block; **(B)** The local anesthetic diffuses within the lumbar interfascial triangle. QL, quadratus lumborum; PM, psoas major; LD, latissimus dorsi; ES, erector spinae; LIFT, lumbar interfascial triangle.

The target of the block is the lumbar interfascial triangle (LIFT) behind the quadratus lumborum. LIFT consists of the lateral border of the erector spinae, the PRS, the middle layer of the TLF, the posterior layer of the TLF, and the lateral raphe. A 22-gauge, 80-mm puncture needle (disposable injection needle, Kindly Medical Devices, Zhejiang, China) was inserted into the LIFT using the in-plane technique under ultrasound guidance (**Figure 1A**). After negative pressure suction, the anesthesiologist gave 0.5 ml of isotonic saline for water separation. Subsequently, 0.375% ropivacaine 30 ml or isotonic saline 30 ml was given in the LIFT for the blockade. The success of the block was judged by the effective diffusion of local anesthetic in the LIFT (**Figure 1B**). All nerve blocks were performed by a skilled anesthesiologist (JC) who had performed over 300 quadratus lumborum blocks. The patient underwent continuous monitoring of vital signs in the nerve block room. Thirty minutes after the block was completed, the patient was transported to the operating room.

### General anesthesia procedure

Patients had a three-lead electrocardiogram, measurement of pulse oxygen saturation, and noninvasive blood pressure monitoring upon admission to the operating room. Lactated Ringer’s solution was instilled through peripheral intravenous access. General anesthesia was performed by intravenous injection of 0.35 μg/kg sufentanil, 2 mg/kg propofol, and 0.7 mg/kg rocuronium sequentially. The anesthesiologist completed the tracheal intubation assisted by video laryngoscope, and the tidal volume was set to 6−8 ml/kg, and the ventilation frequency was 12−15 bpm. The depth of anesthesia was maintained by the intravenous-inhalation combined anesthesia during the operation, which included continuous inhalation of 40% O_2_, 60% N_2_O, and 1.5−2.0% sevoflurane to maintain the MAC value between 1.1−1.3, and continuous infusion of remifentanil at a starting rate of 0.01 μg/kg/min. When blood pressure increased 20% from the baseline value, 0.1 μg/kg sufentanil was injected, and the infusion speed of remifentanil was adjusted, but the maximum dose did not exceed 1 μg/kg/min. The depth of muscle relaxation was maintained with 1/4 the induction dose of the muscle relaxant. All patients received an intraoperative infusion of lactated Ringer’s solution to maintain blood volume, and the infusion rate was 10 ml/kg/h. If blood pressure was 20% below baseline, the anesthesiologist adjusted the depth of anesthesia or administered an appropriate dose of vasoactive drugs. Patients were transfused with suspended red blood cells based on blood loss and hemoglobin values. Thirty minutes before the end of the operation, 0.1 μg/kg sufentanil was intravenously injected for prophylactic analgesia, and other analgesic drugs were not given after that. When the skin was sutured, the inhalation of sevoflurane and the infusion of remifentanil were stopped. After patients returned to the supine position, the inhalation of N_2_O was arrested. After patients were conscious, spontaneous breathing recovered, and muscle strength reached grade III-IV (III [the upper arm could be lifted off the bed surface but did not resist resistance], IV [the upper arm could resist resistance but not wholly]), the tracheal tube was removed and sent to the post-anesthesia care unit (PACU).

### Sutureless laparoscopic partial nephrectomy procedure

**Figure 2:**
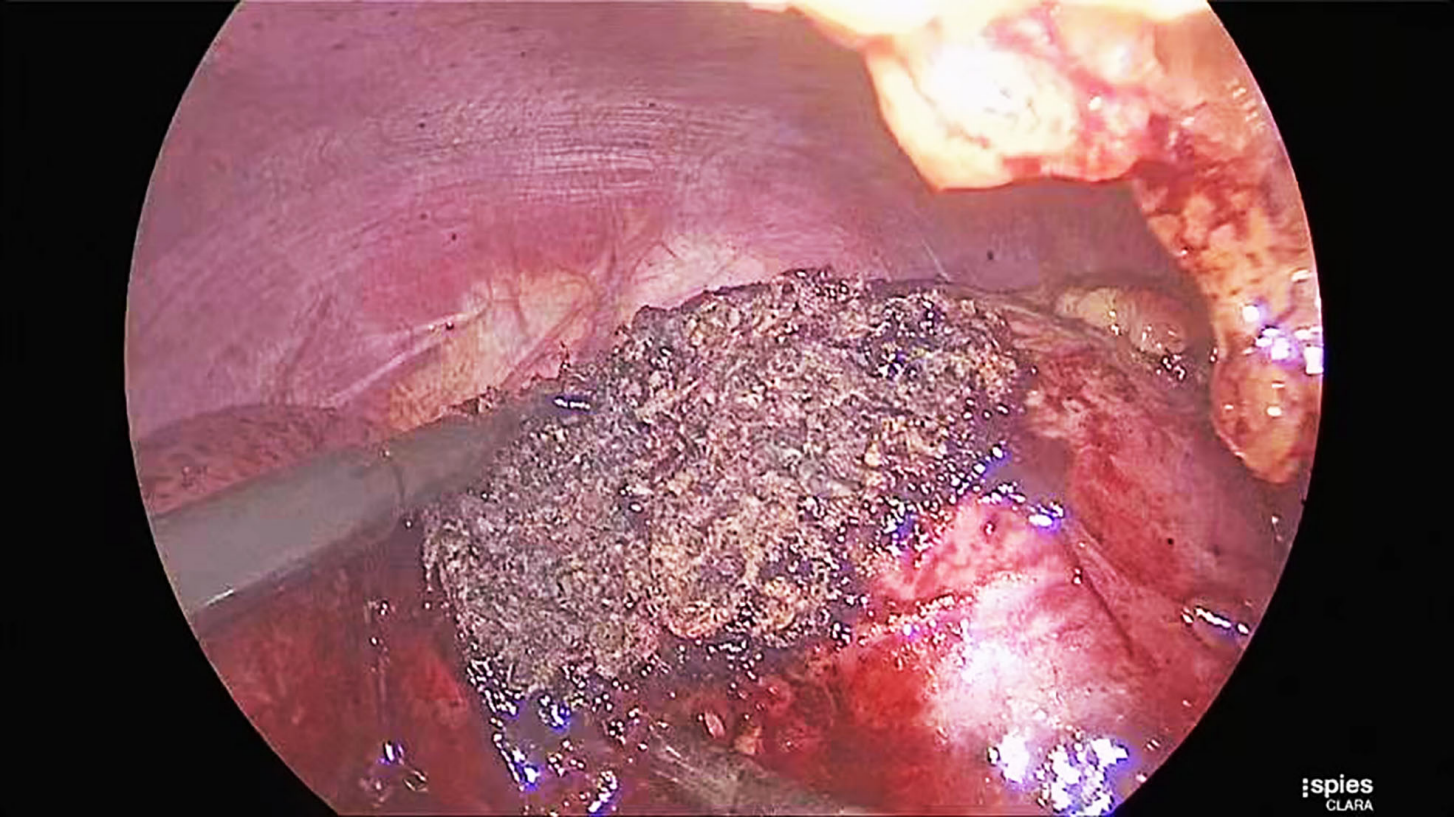
Repeated coagulation to form a helmet-like eschar helped maintain hemostasis.

Patients were placed in the lateral decubitus position. Surgeons used a three-cannula technique with a transperitoneal or retroperitoneal approach. After entering the retroperitoneal space, the surgeons opened Gerota’s fascia and perirenal fat to identify the tumor’s location. The renal hilum was isolated to prevent catastrophic bleeding when the cancer was more extensive than 3 cm in diameter. The renal parenchyma surrounding the tumor was marked by monopolar coagulation. Tumors were excised with cold scissors, dissected directly, and enucleated with a vacuum-assisted aspirator outside the pseudocapsule. When bleeding vessels were observed, a monopolar hook was used for coagulation in spray mode. After tumor resection was completed, monopolar coagulation was repeated on the tumor bed in spray mode (100 W) and fulgurate mode (60 W) through a monopolar hook (**Figure 2**). A drainage tube was placed near the tumor bed. The resected tumor was removed through a laparoscopic retrieval bag.

### Postoperative analgesia procedure

As soon as patients arrived in the PACU, the PCIA device (Electronic micropump, FORNIA Medical Devices, Zhuhai, China) was connected and activated by the anesthesia nurse. The PCIA device contained 2 ug/kg sufentanil and 0.3 mg ramosetron and isotonic saline, totaling 100 ml. The parameters of the PCIA device were set to a background dose of 1 ml/h, a single compression dose of 2 ml, a locking time of 15 min, and a maximum dose of 7 ml/h. In the PACU, an anesthesia nurse assisted patients with compressions on PCIA devices when the patients’ pain scores (Numerical Rating Scale, NRS) were assessed as ≥4. After discharge from the PACU, all patients received flurbiprofen 50 mg and axetil administered at 24-hour intervals. Patients received 5 mg IV morphine as rescue analgesia at 10-min intervals until NRS score <3, either in the PACU or surgical ward.

### Blood glucose and cortisol monitoring procedures

The nurses assigned to care for patients used vacuum blood collection tubes to draw 3 ml of venous blood through venous access when patients entered the operating room and remained in the PACU for 30 min. The collected blood samples were immediately sent to the Laboratory Department of Shengjing Hospital of China Medical University to determine cortisol and blood glucose concentrations. The laboratory physician checked the patient’s test results and uploaded them to the laboratory information management system (LIS). Data on blood glucose and cortisol concentrations were collected by two anesthesiologists (SL and XG) independent of the study and who were blinded to the grouping of patients and the intervention they received.

### Outcomes

The primary outcome measures were the total consumption of sufentanil within 12 h and the 15-item quality of postoperative recovery (QoR-15) scores at 48 h after surgery (the time to reach the PACU was defined as 0 h). Secondary outcome measures were (1) NRS at rest (NRSr) and movement (NRSm) after surgery (rest was defined as supine position and movement was defined as six alternate leg lifts off the bed) (2), sufentanil consumption and number of patients requiring additional rescue analgesia (3), the time of the first compression of the analgesic pump (the time from patient’s arrival at the PACU to first compression of PCIA device [min]) (4), blood sugar and cortisol concentrations (5), the time of the patient’s first excretion, ambulation, and drainage tube and urinary catheter removal (the time from patient’s arrival in the PACU to event occurrence [d]), and the number of days of hospitalization after surgery (the time from patient’s arrival in the PACU to discharge [d]) (6), adverse events such as nausea, vomiting, hypotension (blood pressure below 30% of baseline value), bradycardia (heart rate below 60 bpm), excessive sedation (patient slow or unresponsive to loud stimuli), femoral nerve block (decreased quadriceps muscle strength, manifested as lower extremity weakness or falls), pruritus, or generalized local anesthetic toxicity.

### Assessment of outcomes

Resting and movement pain scores were assessed and recorded using a numerical rating scale (0 no pain, 1−3 mild pain, 4−6 moderate pain, 7−9 severe pain, 10 unbearable pain) by an anesthesia nurse blinded to patient grouping and intervention at 0 h postoperatively. In the surgical ward, nurses assessed and recorded pain at rest and movement using a numerical rating scale at prespecified time points (6 h, 12 h, 24 h, and 48 h). The nurses involved in the assessment were blinded to the grouping of patients and the interventions they received. The PCIA device recorded the cumulative consumption of sufentanil within 12 h, 24 h, 48 h, and consumption of sufentanil in different periods (0−2 h, 2−6 h, 6−12 h, 12−24 h, 24−48 h) after surgery and the time of first pressing the analgesic pump. The Physician Management Station of the Hospital Information System (DHIS) recorded the number of patients requiring rescue analgesia. The Nurse Management Station of the Hospital Information System (NHIS) recorded the time of the patient’s first excretion, ambulation, drainage tube, urinary catheter removal, and the number of days of hospitalization after surgery. Two independent anesthesiologists (XG and SL) collected data blinded to the grouping of patients and the interventions they received.

### Statistics and sample size

The cumulative consumption of sufentanil within 12 h after surgery and the QoR-15 scores at 48 h after surgery were the observed primary outcome, and the sample size was calculated based on these. In our retrospective study of 20 patients who underwent sLPN in the past one month, the mean consumption (standard deviation, SD) of sufentanil for PCIA within 12 h after surgery was 28.76 (8.97) μg, the mean score (standard deviation, SD) of QoR-15 scores at 48 h after surgery was 92.12 (10.05). With background doses, we expected to detect at least a 30% reduction in cumulative consumption of sufentanil in the intervention group compared to the control group within 12 h after surgery, significance level (α) = 0.05, power = 90% (1-β). The sample size of the intervention group N_1_ = 24 and the control group N_2_ = 24 was calculated using the Power Analysis and Sample Size 15.0 software. A change of 10 for the QoR-15 scores was considered to represent a clinically relevant difference based on clinical experience and expert advice. At the same significance level and power, the sample size for each group calculated by the QoR-15 scores was 23. Therefore, the sample size calculated by the cumulative consumption of sufentanil within 12 h after surgery was used as the final result. Considering the potential dropout (20%), at least 60 patients (30 patients in each group) were finally included to decrease power loss.

Data were analyzed using Statistical Package for Social Science 25.0 software. All data analysis was performed according to a prespecified plan. The Shapiro-Wilk test was used to test the normality of continuous variables, expressed as means and SDs if they were normally distributed and as medians and interquartile ranges if they were non-normally distributed. Baseline and surgical characteristics between the intervention and control groups were analyzed by *t*-tests, Mann-Whitney U test, or chi-square test. Cumulative sufentanil consumption within 12 h, 24 h, and 48 h and QoR-15 scores at 48 h after surgery were assessed using the Mann-Whitney U test and the Hodges-Lehmann method to estimate pseudo-medians difference and 95% CI. Correlations between cumulative sufentanil consumption within 12 h and QoR-15 scores were analyzed using rank correlation. Repeated-measures data (the NRS and the sufentanil consumption) were assessed using generalized estimating equations (GEE) with an identification number (ID) as the primary variable, time as the main within-variable, and NRS and sufentanil consumption as dependent variables. The time of first pressing the analgesic pump within 48 h after the surgery was analyzed by Kaplan-Meier survival curve and log-rank tests. Hazard ratios and 95% CI were estimated using univariate COX regression. The blood glucose and cortisol concentrations between the intervention and control groups were analyzed by covariance (ANCOVA) analysis, and the least-squares mean (LSM) difference and 95% CI were estimated. Categorical data were expressed as the number of cases (percentage) and analyzed using the chi-square test, continuous corrected chi-square test, or Fisher’s exact test. All statistical tests were two-sided, and a *p-*value less than 0.05 was considered statistically significant.

## Results

**Figure 3:**
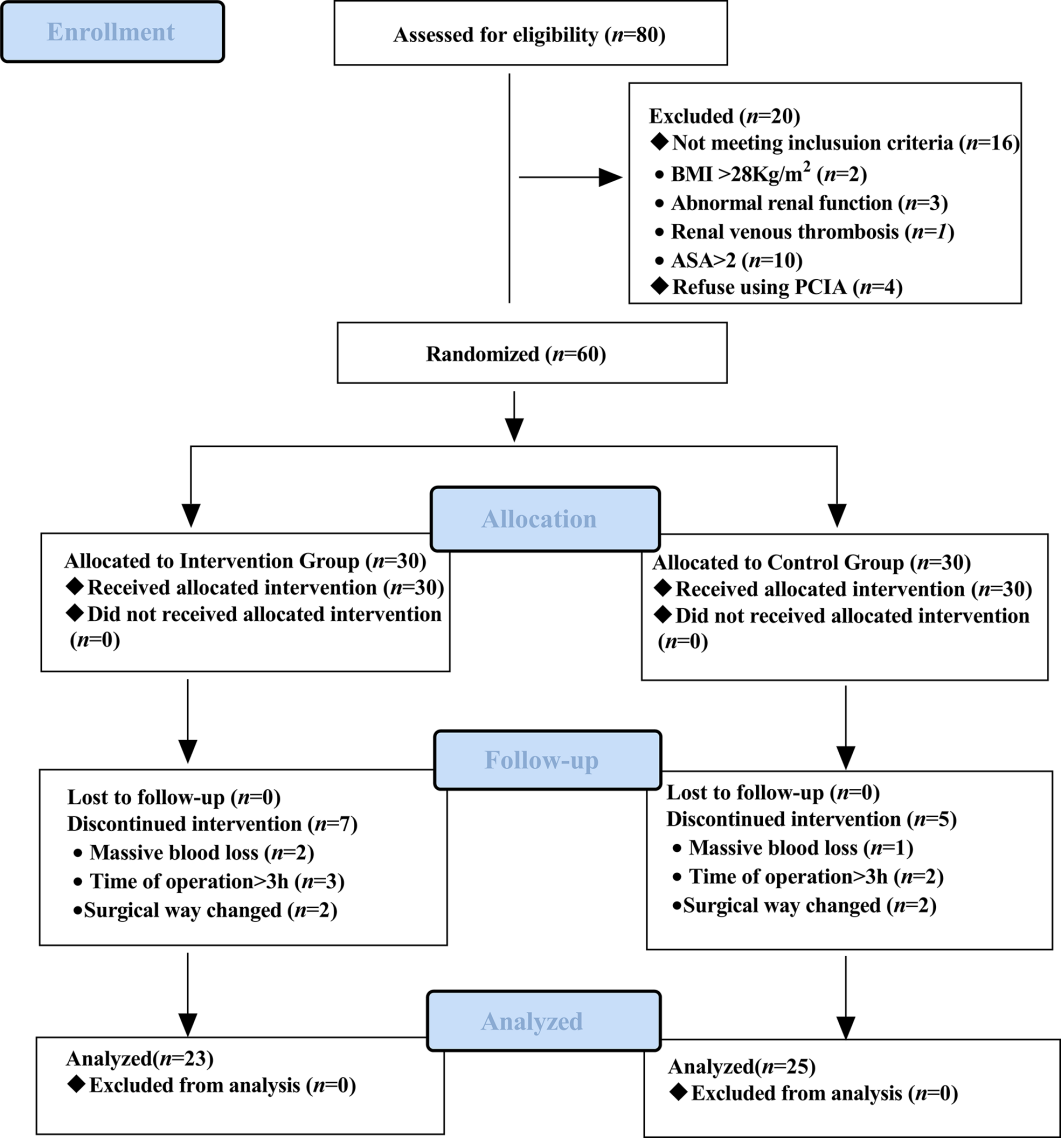
Consolidated Standards of Reporting Trials (CONSORT) flow diagram.

The recruitment time of the source population was from November 2021 to April 2022, and the location was Shengjing Hospital of China Medical University. Of the 80 eligible participants, 60 were included and randomly assigned to the intervention group (*n* = 30) and the control group (*n* = 30). Sixty patients received the intervention in the control and intervention groups, but 12 discontinued the intervention during the study. In the intervention group, two patients had their surgical procedure changed, two underwent blood transfusions due to massive blood loss during surgery, and three patients had an operative time of more than 3 h. In the control group, two patients had their surgical procedure changed, one underwent a blood transfusion for massive intraoperative blood loss, and two patients had an operative time of more than 3 h. Forty-eight patients completed the study as pre-protocol and were included in the analysis of pre-protocol (i.e., 23 in the intervention group and 25 in the control group). The diagram details the flow of patients through the trial (**Figure 3**). Baseline characteristics of the two groups of patients were comparable (**Table 1**).

**Table 1:** Baseline and surgical characteristics.

	Control group(*n* = 25)	Intervention group(*n* = 23)	*p*
Age (year), medians (range)	57.0 (46.0 to 61.0)	53.0 (43.0 to 58.0)	0.420
Sex, number			
Male	13	13	0.753
Female	13	10	
Height (cm), means ± SDs	168.7 ± 7.3	169.6 ± 7.3	0.662
Weight (kg), means ± SDs	69.8 ± 8.8	69.4 ± 7.9	0.865
BMI (kg/m^2^), means ± SDs	24.4 ± 2.1	24.3 ± 1.7	0.867
ASA physical status, number			
I	8	6	0.653
II	17	17	
Surgical approach, number			
Transperitoneal	12	9	0.536
Retroperitoneal	13	12	
Operation time (min), means ± SDs	138.6 ± 30.2	125.9 ± 35.3	0.185

BMI, Body mass index; ASA, American Society of Anesthesiologists classification.

### Primary outcomes

**Figure 4:**
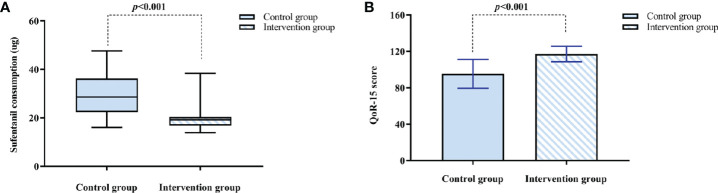
**(A)** Sufentanil consumption within 12 h after surgery. The results are reported as median (IQR). **(B)** The QoR-15 score at 48 h after surgery. The results are reported as means and SDs. The time to reach PACU is defined as 0 h. QoR-15: 15-item quality of postoperative recovery.

The intervention group had lower cumulative consumption of sufentanil within 12 h after surgery compared with the control group (19.20 [16.80 to 20.40] vs. 28.60 [22.40 to 36.30], *p* < 0.001) (**Figure 4A**), and the pseudo-median difference and 95% CI was 9.40 (6.36 to 12.80). The intervention group had higher QoR-15 scores at 48 h postoperatively compared to the control group [(117.17 ± 8.48 vs. 95.36 ± 15.88), *p* < 0.001] (**Figure 4B**), and the pseudo-median difference and 95% CI was 21.00 (15.00 to 28.00). There was a significant correlation between cumulative consumption of sufentanil within 12 h and QoR-15 scores at 48 h postoperatively (r_s_ = −0.750, *p* < 0.001).

### Secondary outcomes

**Figure 5:**
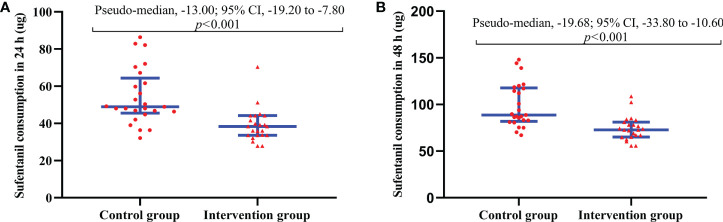
**(A)** Total sufentanil consumption within 24 h after surgery. **(B)** Total sufentanil consumption within 48 h after surgery. The results are reported as median (IQR). The time to reach PACU is defined as 0 h. The Hodges-Lehmann method was used to estimate pseudo-medians difference and 95% CI.

The NRSr of the intervention group was lower at 0 h, 6 h and 12 h after surgery compared with the control group (Difference [95% CI]: −1.26 [−1.79 to −0.72], −0.84 [−1.35 to −0.33], −0.58 [−1.05 to −0.11], *p* < 0.05) (**Table 2**). There was no significant difference in the NRSr between the intervention group and the control group at 24 h and 48 h after surgery (*p* > 0.05). The intervention group had lower NRSm at 0 h, 6 h and 12 h postoperatively compared with the control group (Difference [95% CI]: −1.96 [−2.59 to −1.32], −1.47 [−2.10 to −0.83], −0.75 [−1.31 to −0.19], *p* < 0.05) (**Table 2**). There was no significant difference in the NRSm between the intervention group and the control group at 24 h and 48 h after surgery (*p* > 0.05). At prespecified intervals (0 to 2 h, 2 to 6 h, 6 to 12 h, 12 to 24 h, and 24 to 48 h) after surgery, the intervention group had lower consumption of sufentanil compared with the control group (Difference [95% CI]: −3.14 [−4.35 to −1.94], −4.23 [−5.88 to −2.59], −3.47 [−5.62 to −1.31], −4.38 [−7.73 to −1.03], −8.72 [−14.69 to −2.74], *p* < 0.05) (**Table 2**). The consumption of sufentanil within 24 h in the intervention group was lower compared with the control group (38.40 [33.60 to 44.20] vs. 48.96 [45.58 to 64.40], *p* < 0.001), the pseudo-median difference and 95% CI was −13.00 (−19.20 to −7.80) (**Figure 5A**). The consumption of sufentanil within 48 h in the intervention group was lower compared with the control group (72.80 [65.00 to 81.00] vs. 88.80 [81.98 to 117.78], *p* < 0.001), the pseudo-median difference and 95% CI was −19.68 (−33.80 to −10.60) (**Figure 5B**).

**Table 2:** The NRS scores at different time points and sufentanil consumption at different periods.

Variable	Control group	Intervention group	Difference (95% CI)	Wald	*p* ^#^
NRSr, medians (range)					
0h	2.0 (1.0 to 3.0)	1.0 (0.0 to 1.0)	−1.26 (−1.79 to −0.72)	21.338	<0.001
6h	2.0 (1.0 to 3.0)	1.0 (1.0 to 1.0)	−0.84 (−1.35 to −0.33)	10.389	0.001
12h	2.0 (1.0 to 3.0)	1.0 (1.0 to 2.0)	−0.58 (−1.05 to −0.11)	5.934	0.015
24h	2.0 (1.0 to 3.0)	2.0 (1.0 to 2.0)	−0.15 (−0.73 to 0.44)	0.245	0.621
48h	2.0 (1.0 to 2.5)	2.0 (1.0 to 3.0)	0.03 (−0.55 to 0.60)	0.008	0.929
NRSm, medians (range)					
0h	5.0 (5.0 to 6.0)	3.0 (3.0 to 4.0)	−1.96 (−2.59 to −1.32)	36.557	<0.001
6h	5.0 (4.0 to 6.0)	4.0 (3.0 to 4.0)	−1.47 (−2.10 to −0.83)	20.493	<0.001
12h	5.0 (4.0 to 6.0)	4.0 (4.0 to 5.0)	−0.75 (−1.31 to −0.19)	6.986	0.008
24h	4.0 (4.0 to 5.0)	4.0 (3.0 to 5.0)	−0.30 (−0.98 to 0.38)	0.736	0.391
48h	4.0 (4.0 to 5.0)	4.0 (3.0 to 5.0)	−0.48 (−1.31 to 0.35)	1.270	0.260
Sufentanil (μg), medians (range)					
0 to 2h	6.0 (4.0 to 8.3)	2.8 (2.6 to 3.2)	−3.14 (−4.35 to −1.94)	26.041	<0.001
2 to 6h	9.8 (6.3 to 14.2)	5.6 (5.1 to 6.4)	−4.23 (−5.88 to −2.59)	25.513	<0.001
6 to 12h	14.3 (9.0 to 17.9)	9.6 (8.0 to 11.4)	−3.47 (−5.62 to −1.31)	9.955	0.002
12 to 24h	21.0 (18.9 to 30.0)	19.2 (16.8 to 22.4)	−4.38 (−7.73 to −1.03)	6.581	0.010
24 to 48h	38.8 (35.4 to 50.8)	33.8 (31.2 to 38.4)	−8.72 (−14.69 to −2.74)	8.163	0.004

NRSr, numerical rating scale at resting; NRSm, numerical rating scale at movement; #, individual effects of interventions were calculated by generalized estimating equations (GEE).

**Figure 6:**
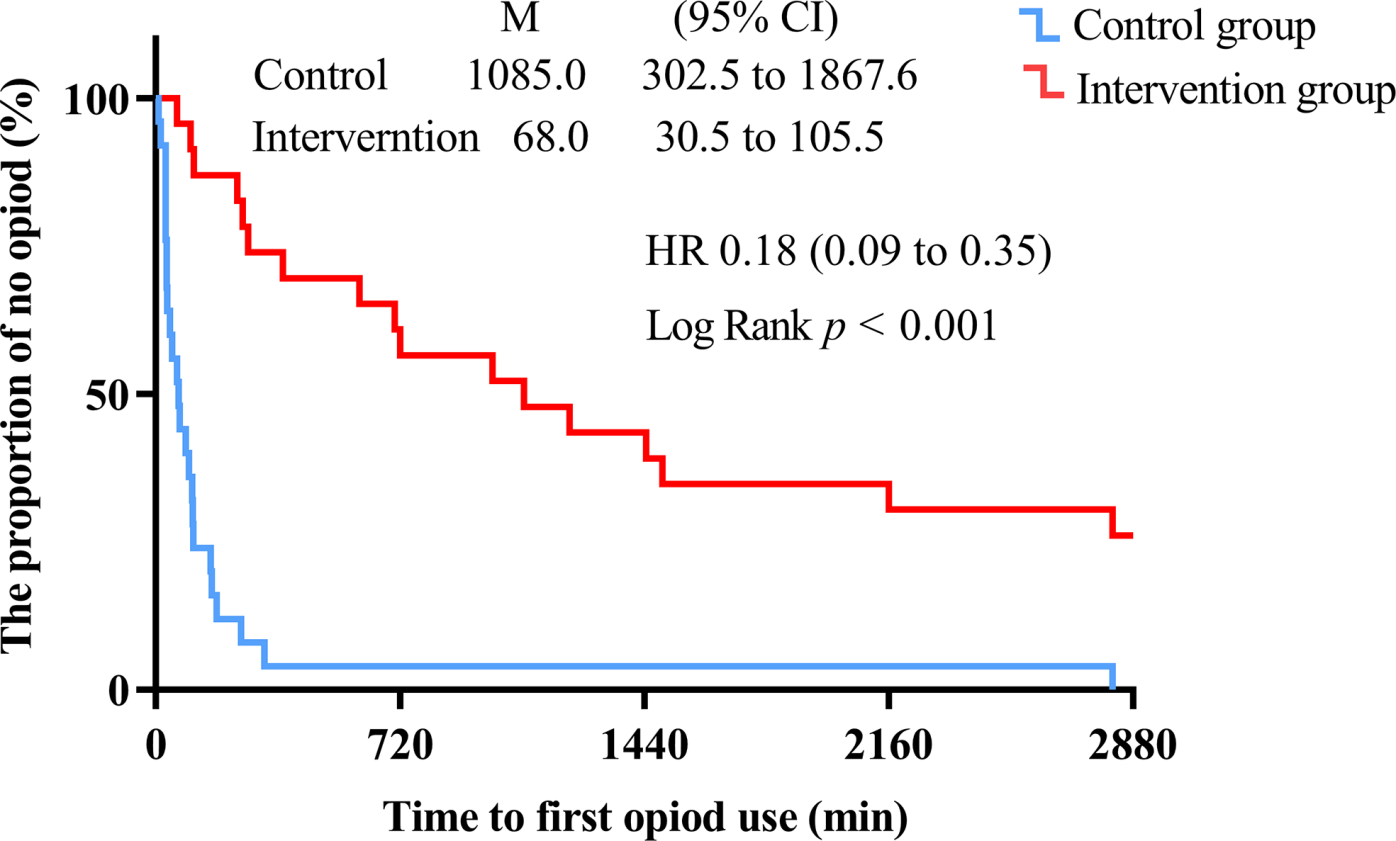
Kaplan-Meier survival plot of time to the first compression of the analgesic pump within 48 h after surgery (min). The time to reach PACU is defined as 0 h. Hazard ratios (HR) and 95% CI were estimated using univariate COX regression.

**Figure 7:**
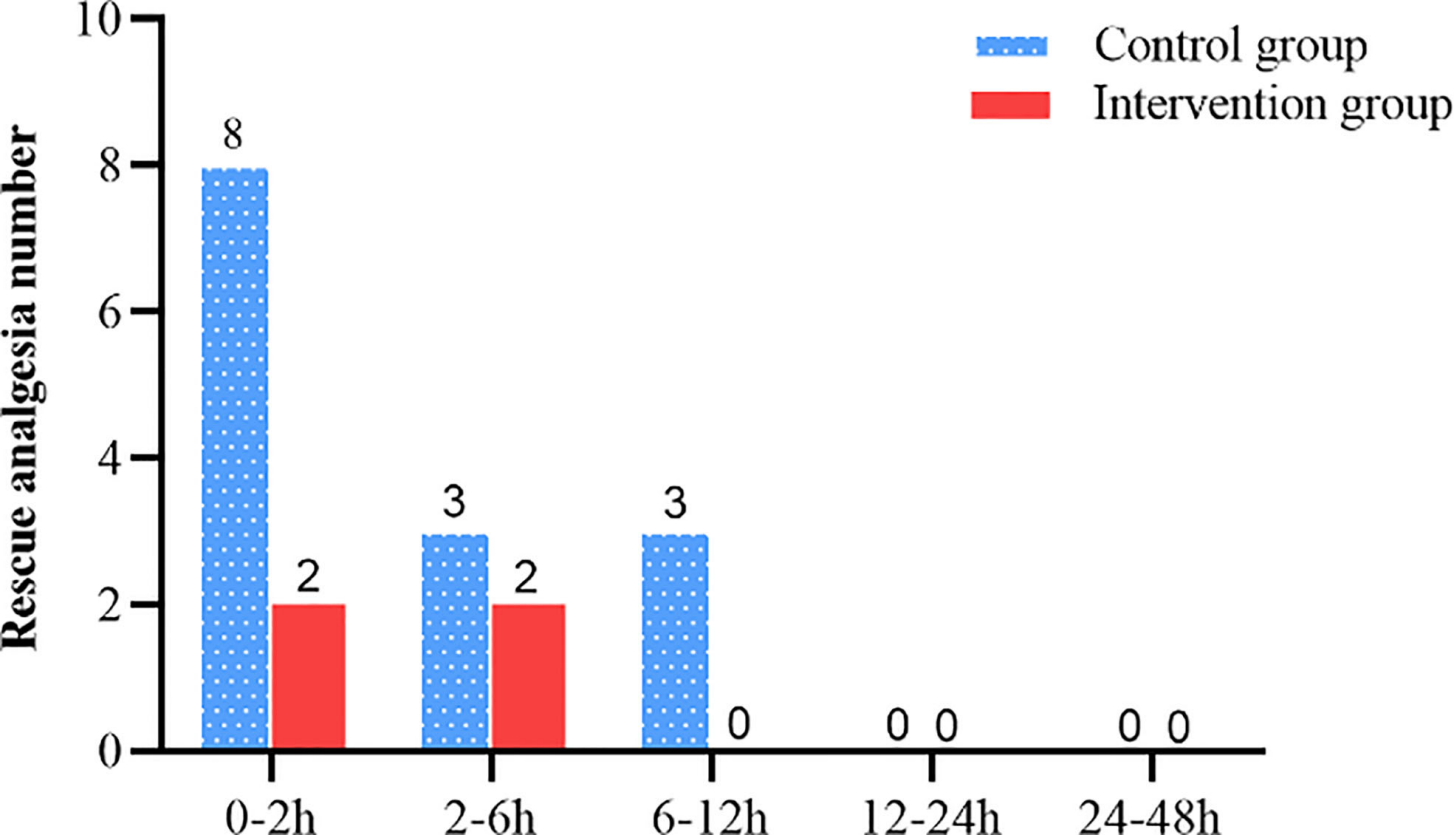
The number of people requiring additional rescue analgesia.

The intervention group took longer to press the analgesic pump for the first time within 48 h after surgery compared with the control group (1085.0 [302.5 to 1867.6] vs. 68.0 [30.5 to 105.5], Log-Rank *p* < 0.001) (**Figure 6**). The hazard ratio (HR) and 95% CI for the intervention group and the control group were 0.18 (0.09 to 0.35). There was no significant difference between the intervention and control groups in the number of patients requiring rescue analgesia within 48 h after surgery (*p* > 0.05) (**Figure 7**).

**Figure 8:**
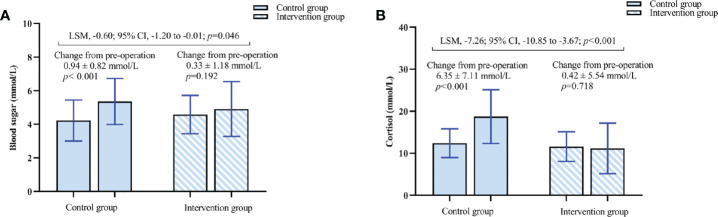
**(A)** Changes in blood sugar concentration. **(B)** Changes in cortisol concentration. The results are reported as means and SDs. The Least Squares Mean (LSM) difference and 95% CI were estimated by analysis of covariance (ANCOVA).

The postoperative blood glucose concentration in the intervention group was lower than that in the control group (4.91 ± 1.63 vs. 5.36 ± 1.37, *p* = 0.046) (**Table 3**, **Figure 8A**). The LSM difference and 95% CI between the two groups was −0.60 (−1.20 to −0.01). The postoperative cortisol concentration in the intervention group was lower than that in the control group (11.17 ± 6.01 vs. 18.75 ± 6.40, *p* < 0.001) (**Table 3**, **Figure 8B**). The LSM difference and 95% CI between the two groups was −7.26 (−10.85 to −3.67).

**Table 3:** Blood sugar and cortisol concentrations.

Blood sugar, mmol/L	N	Pre-operation	Postoperation	LSM 95% CI^#^	*F*	*p*
Control group	25	4.42±1.22	5.36±1.37	−0.60 (−1.20 to −0.01)	4.199	0.046
Intervention group	23	4.58±1.14	4.91±1.63			
Cortisol, mmol/L						
Control group	25	12.40±3.40	18.75±6.40	−7.26 (−10.85 to −3.67)	16.576	<0.001
Intervention group	23	11.59±3.52	11.17±6.01			

LSM, Least Squares Mean Difference; # effect size was calculated by analysis of covariance.

The time to first excretion, time to first ambulation and discharge were shorter in the intervention group compared with the control group (2.0 [2.0 to 3.0] vs 3.0 [3.0 to 5.0], 2.0 [1.0 to 3.0] vs 4.0 [2.0 to 5.5], 6.0 [5.0 to 7.0] vs 7.0 [5.5 to 8.5], *p* < 0.05) (**Table 4**). There was no statistically significant difference between the intervention and control groups in the postoperative urinary catheter and drainage catheter removal time (*p* > 0.05). There were no significant differences in nausea, vomiting, respiratory depression, excessive sedation, hypotension, or bradycardia between the intervention group and the control group (*p* > 0.05). Femoral nerve block and local anesthetic intoxication were not observed in both intervention and control groups.

**Table 4:** Early postoperative recovery and adverse events.

Early recovery	Control group (*n* = 25)	Intervention group (*n* = 23)	*p*
Excretion, d	3.0 (2.0 to 5.0)	2.0 (2.0 to 3.0)	0.006
Ambulation, d	4.0 (2.0 to 5.5)	2.0 (1.0 to 3.0)	<0.001
Catheter removal, d	5.0 (3.0 to 7.0)	4.0 (3.0 to 5.0)	0.079
Drain removal, d	6.0 (5.0 to 7.5)	5.0 (4.0 to 6.0)	0.123
Discharge, d	7.0 (5.5 to 8.5)	6.0 (5.0 to 7.0)	0.041
Adverse event, No. (%)			
Nausea	6 (24.0)	4 (17.4)	0.836^#^
Vomit	3 (12.0)	0	0.263^#^
Respiratory Depression	1 (4.0)	0	1.000^*^
Excessive sedation	1 (4.0)	0	1.000^*^
Hypotension	3 (12.0)	0	0.263^#^
Bradycardia	1 (4.0)	0	1.000^*^

# Continuous corrected chi-square test, * Fisher’s exact test

## Discussion

This is the first randomized, controlled, triple-blind study to report the application of posterior QLB in sLNP. We found that patients who received posterior QLB had significantly lower opioid consumption within 12 h postoperatively and had a better quality of recovery at 48 h postoperatively. Second, we found that patients who received posterior QLB had significantly lower NRS at resting and movement within 12 h after surgery, substantially more extended time to the first compression of the analgesic pump, and an effectively controlled stress response. At the same time, we found that patients who received posterior QLB had earlier recovery of gastrointestinal function and significantly shorter time to ambulation and hospital discharge.

In this trial, we found that for patients receiving sLNP, the use of QLB reduced opioid consumption by 36.1% in the intervention group compared to the control group within 12 h after surgery, which is consistent with our minimum pre-trial expectations (30%). However, our reduction was more conservative compared to the study by Dam et al. (11), which showed a 43% reduction in opioid consumption in the intervention group compared to the control group within 12 h after surgery. However, it should be emphasized that the QLB performed by Dam et al. (11) was bilateral, while our intervention was a unilateral block only for the surgical side, which may make the final analgesic effect somewhat different. Moreover, Dam et al. (11) performed anterior QLB, which is a different approach than posterior QLB. It should be noted that the different puncture paths of QLB will affect the diffusion of local anesthetics (15). The anterior QLB allows for more paraspinal distribution of local drugs than the lateral and posterior QLB (16). Secondly, the postoperative analgesia pump was set to a background dose of 1 ml/h, which means that a specific dosage of opioids will be consumed even if the patient experiences no or only mild pain after surgery. The presence of background doses will cause false-negative interference with the actual results of the intervention group, masking inter-individual differences (17).

Interestingly, Li et al. (12) found that although the posterior QLB approach has a relatively apparent postoperative analgesic effect, there was no statistically significant difference in opioid consumption compared with the control group, which contradicted our research results. The possible reason is that Li et al. (12) tested the subjects on the block plane after the QLB procedure, which violated the principle of blinding from the subject’s perspective. Patients who know they have undergone QLB may be more inclined to reduce opioid use. Our research team strictly followed the triple-blind principle throughout the entire trial process for the objective and scientific nature of the trial results.

We demonstrated for the first time that the application of unilateral posterior QLB block in patients undergoing sLPN could significantly reduce postoperative opioid consumption and improve the quality of patients’ recovery at 48 h after surgery. Our study shows a robust negative relationship between opioid consumption and the quality of recovery, implying that high opioid use may adversely affect patients’ postoperative recovery. Meouchy et al. (18) pointed out that using bilateral lateral QLB in abdominoplasty can reduce postoperative opioid consumption and improve the quality of postoperative recovery. Kwak et al. (10) pointed out that using a single lateral QLB in laparoscopic partial nephrectomy can reduce postoperative opioid consumption, but the quality of recovery score at 48 h after surgery was not significantly different from that of the control group. Xia et al. (19) showed that using a single anterior QLB in knee arthroplasty significantly reduced postoperative opioid consumption and improved postoperative recovery scores. This discrepancy in results is most likely due to different approaches of QLB and local anesthetic doses. Our study supports the effect of posterior QLB on the quality of recovery in patients. We used QoR-15 to evaluate the quality of postoperative recovery of patients. This scale is more concise than QoR-40, with unnecessary content removed. The postoperative psychological state, pain level, self-care level, and adverse reactions of patients were converted into specific values, and the clinical feasibility and evaluation objectivity were better than that of QoR-40.

In this trial, we found that all patients receiving posterior QLB had significantly lower NRS scores at rest and movement within 12 hours after surgery than the control group. Li et al. (12) performed unilateral posterior QLB in patients undergoing laparoscopic nephrectomy. They found that the intervention group presented with mild pain at rest and movement 24 h postoperatively, and pain scores were significantly lower than those in the control group. Blanco and Ökmen et al. (20, 21) performed bilateral posterior QLB in patients before surgery, and their results also confirmed that posterior QLB has a better analgesic effect. However, our results differed from theirs: the NRS score was higher at movement in the intervention group (median NRS score = 4 between 6 h and 48 h postoperatively). Dam et al. (11) proposed a bilateral QLB block for better somatic and visceral analgesia during laparoscopic partial nephrectomy. Considering that patients undergoing sLPN may experience more severe visceral pain due to extensive intraoperative coagulation impairment, we believe that bilateral posterior QLB block may be more effective for analgesia in sLPN. Our future research will further support this hypothesis. Second, we think that the definition of movement status and the subjectivity of NRS scores may also affect trial results. To further verify the analgesic effect of posterior QLB, we compared the opioid consumption of the two groups in five time periods after surgery. We found that the opioid consumption of the intervention group was significantly lower than that of the control group in each period. Therefore, we believe that posterior QLB block has an analgesic effect on postoperative pain in patients undergoing sLPN.

We also found that posterior QLB significantly prolonged the time to the first opioid requirement. The median time to the first compression of the analgesic pump in the intervention group was significantly longer than in the control group, about 18 h. In the trials of Li et al. (12) and Ahmed et al. (22), the median time to the first compression of the analgesic pump was 13.5 h and 12 h, respectively. This difference may be related to multimodal analgesia’s type or the dose of other analgesics. Prolonging the analgesic duration of QLB is favorable for patients. Several studies (23–25) have shown that various adjuvants (such as dexmedetomidine, dexamethasone) or intravenous drugs (lidocaine, ketamine) used in conjunction with QLB can prolong the analgesic time of QLB, and the placement of catheters for sustained analgesia is also an option (26). However, the mechanisms behind them and their broad clinical applications still require further exploration in the future. It is also worth noting that 6 (26%) patients in the intervention group did not press the analgesic pump postoperatively, while all patients in the control group did. This result suggests that a postoperative analgesic regimen involving posterior QLB will benefit from weaning off opioid dependence in perioperative pain management. This aligns with the international search for an opioid-free or opioid-minimized anesthesia regimen. Reducing long-acting opioids after surgery is significant because it reduces the risk of opioid addiction, opioid-related adverse events, and immunosuppression (27).

Ultrasound-guided QLB is a trunk nerve block technique that has emerged recently. It blocks somatic and visceral pain, and its effect and duration are better than other trunk nerve-block techniques, such as the transverse abdominis plane block (14). According to the anatomical position of the needle tip relative to the quadratus lumborum muscle and the needle insertion trajectory. El-Boghdadly et al. (28), 2016, conducted an anatomical study and nomenclature of multiple approaches to QLB. In our trial, we chose the posterior QLB technique. The main reasons are (1): The anatomical research and mechanism of posterior QLB are still unclear, and it is the most controversial among the four techniques. Its specific injection targets are not unified (LIFT, posterolateral, posteromedial) (14, 29, 30). Moreover, published clinical studies do not specify the injection target of posterior QLB in detail, thus requiring speculation based on the specific operation steps or ultrasound images. However, our trial identified a particular target for injection of posterior QLB, the lumbar fascial triangle. This is also consistent with the posterior QLB optimal point previously proposed by Blanco et al. (14) (2); We believe that the anterior QLB procedure is too close to the peritoneum, which can easily penetrate the peritoneum and cause intestinal damage. Moreover, the target of the anterior QLB block is the anterior TLF between the quadratus lumborum and the psoas muscle. If the needle is inserted too deeply, it is easy to cause lumbar plexus block and lead to complications of lower extremity muscle weakness. However, the research about the clinical safety of QLB technology is limited to a small number of case reports (with hematoma, urinary retention, and muscle weakness) (31–33) (3). The injection targets of the lateral QLB and transverse abdominal fascia block are almost the same, and there is no essential difference between the two block techniques. Furthermore, MRI scans showed that posterior QLB stained more broadly than lateral QLB and provided more predictable local anesthetic spread in the paravertebral space (34). Intramuscular QLB has a specific target location, and cadaver reports (35) do not indicate the spread of the stain to the thoracic paravertebral body. Still, it produced ipsilateral analgesic effects in healthy volunteers. It creates an area of the sensory block from the ventral to the posterior side of the body (with the axillary line as the center point) and from the skin level of the lateral 8th thoracic vertebra to the proximal lateral thigh. Intramuscular QLB appears to be applicable in laparoscopic partial nephrectomy, and future prospective studies could verify the effectiveness of this technique.

At present, the speculation about the analgesic mechanism of QLB may be attributed to the diffusion of local anesthetic along the quadratus lumborum and TLF into the thoracic paravertebral space (9, 36), but by observing the onset speed and duration of analgesia of QLB, its mechanism of action may not stop there. Benetazzo et al. (37) found that the TLF is rich in the nerve distribution. Tesarz et al. (38) found many mechanoreceptors and a high density of sympathetic nerves distributed on the TLF. The MRI study by Blanco et al. (14) found that the amount of drug reaching the paravertebral space was very small, although the diffusion of the local anesthetic into the paravertebral space along the TLF played an important role. Blanco et al. (14) suggested that mechanoreceptors and high density of sympathetic nerves may be related to the mechanism of action of QLB, and the TLF is the leading site of action of QLB. Therefore, the exact mechanism of QLB is unclear. There are still some doubts about whether the local anesthetic diffuses into the paravertebral space and thus exerts the main analgesic effect (39, 40). The choice of the optimal injection site remains undecided. This has also prompted researchers to conduct more dye injection studies at different blocking targets for QLB on embalmed cadavers.

In 2001, Kehlet et al. (41) first proposed the concept of ERAS, based on evidence-based medicine, through a series of perioperative optimization measures to reduce perioperative stress responses and complications and accelerate the postoperative recovery of patients. Reducing perioperative stress responses is an important goal of ERAS. To our knowledge, this is the first clinical study of the effects of QLB on stress responses. In this study, we hypothesized that preoperative implementation of posterior QLB could reduce the perioperative stress response by inhibiting pain signals’ conduction and ultimately promote patients’ rapid postoperative recovery. Our results suggest that implementing posterior QLB in patients receiving sLNP slows postoperative increases in blood glucose and cortisol concentrations. Elevated blood glucose levels caused by stress responses can lead to delayed wound healing and an increased risk of wound infection in patients. We also found that posterior QLB can facilitate early ambulation, early recovery of gastrointestinal function, and early discharge in patients undergoing sLPN. The results of Zhu et al. (42) also support our conclusion. We did not find a significant difference in side effects caused by opioids between the two groups. In the study by Zhu et al. (42), the incidence of nausea and vomiting in the intervention group was significantly lower than in the control group. We added ramosetron to the analgesic pump, which reduced the incidence of nausea and vomiting in some patients. We have provided scientific and standardized guidance to the subjects and their families on using analgesia pumps before and after surgery. The standardized use of postoperative analgesia pumps will also reduce the occurrence of postoperative nausea and vomiting to a certain extent. In addition, although the consumption of sufentanil in the control group was higher, the maximum dose per h was limited, which may be the reason for the absence of side effects such as excessive sedation and respiratory depression.

Although this study achieved the expected findings, there are still some limitations. To ensure all subjects were blinded, we did not measure the cutaneous sensory block level, and the block’s success was judged by the effective diffusion of local anesthetic in the LIFT. From another perspective, we tried our best to ensure the triple-blind state of operators, subjects, and information collectors to minimize information bias and ensure the authenticity of trial results. We measured the blood glucose and glucocorticoid concentrations only before the operation and 30 minutes after reaching the PACU. Multiple time points in the perioperative period were not calculated, so intraoperative fluctuations and changing trends could not be evaluated. Incisional and deep abdominal pain coexisted after the sLPN, and the two different sources of pain were not assessed separately, which is not conducive to verifying whether QLB can provide visceral analgesia. The conclusion that the sLPN has higher postoperative pain than traditional surgery is based on individual retrospective observational studies, and no published literature shows the same evidence. However, a prospective study at our Renal Surgery Research Center may provide solid evidence for this view in the future. This is a small sample study, and our conclusions may be exploratory rather than confirmatory. However, our study may have implications for developing this new surgical technique.

## Conclusions

Our trial demonstrated that patients who received posterior QLB had significantly lower opioid consumption within 12 h postoperatively and had a better quality of recovery at 48 h postoperatively. Therefore, we recommend posterior QLB as an option for postoperative analgesia in patients undergoing sLPN.

## Data availability statement

The raw data supporting the conclusions of this article will be made available by the authors, without undue reservation.

## Ethics statement

The studies involving human participants were reviewed and approved by The Ethics Committee of Shengjing Hospital of China Medical University. The patients/participants provided their written informed consent to participate in this study.

## Author contributions

Study design/planning: YZ, SQ, ZL, JC. Study conduct: YZ, SQ, ZL, SL, XG, YK, JC. Data analysis: YZ, HX, FS, QZ, LC, WW. Writing paper: YZ, ZL, SQ. Revising paper: YZ, ZL, SQ, HX, FS, QZ, YK, XG, SL, JC, LC, WW. All authors contributed to the article and approved the submitted version.

## Funding

This work was partly supported by a Key Research and Development Project of Liaoning Province in 2020 of the Science and Technology Department of Liaoning Province (Joint Fund Project) grant 2020JH2/10300129.

## Conflict of interest

The authors declare that the research was conducted in the absence of any commercial or financial relationships that could be construed as a potential conflict of interest.

## Publisher’s note

All claims expressed in this article are solely those of the authors and do not necessarily represent those of their affiliated organizations, or those of the publisher, the editors and the reviewers. Any product that may be evaluated in this article, or claim that may be made by its manufacturer, is not guaranteed or endorsed by the publisher.
